# Patenting of Surgical Instruments

**Published:** 1865-10

**Authors:** 


ARTICLE XXXVII.

EDITOR MEDICAL EXAMINER:—

DEAR SIR,—I notice, in the June No. of the EXAMINER, a short article over the signature of X, upon the patenting of surgical instruments, and comparing it to patenting the "Paths of Virtue," by a clergyman.

Now, unfortunately for the comparison, this is just what the clergyman does. Look every week into the *N. Y. Independent*, and see the heading of the sermon of Henry Ward Beecher, "Entered according to Act of Congress," &c. Take up any book written by an American upon religion or morals, and the warning appears against the use of its wisdom without paying for it. The copyright is to literary labor what the patent right is to mechanical labor; and it becomes a question whether it is not time for the medical profession to outgrow its old prejudice against patent rights. This prejudice has lingered from the dark ages; in which to steal the honest earnings of the *worker* was honorable. Now the public maxim is:—"To every man a just reward for his labor."

X admits that the feeling or usage against patents is not one growing out of any necessary relations, but claims that it is one of common consent, among medical men. Then let us, by common consent, have the labor of men who make books without paying them for it. The book maker may console himself with the reflection that he reads a vast amount, which he has never paid for, and besides that he is working for science and humanity. It is one of the strange things that medical men will willingly pay an author through the copyright for his labor, but will not willingly pay an inventor for his labor, though in many cases more valuable to the profession. This unreasonable prejudice is fostered by the general compounding of secret remedies—nostrums which remunerate through a gigantic system of advertising and falsehood with patents. Nobody that understands the business patents a medicine or compound, but he keeps the composition a secret, and protects himself from imitation by the name, which is regarded as a *trade mark*, by the label which may be *copyrighted*, and by the proprietary stamp which is furnished by the Government. The patenting of the compound opens its composition to any one who will pay for transcribing the patent. Z.

